# The Evolution of Clinically Aggressive Triple-Negative Breast Cancer Shows a Large Mutational Diversity and Early Metastasis to Lymph Nodes

**DOI:** 10.3390/cancers13205091

**Published:** 2021-10-12

**Authors:** Héctor Martínez-Gregorio, Ernesto Rojas-Jiménez, Javier César Mejía-Gómez, Clara Díaz-Velásquez, Rosalía Quezada-Urban, Fernando Vallejo-Lecuona, Aldo de la Cruz-Montoya, Fany Iris Porras-Reyes, Víctor Manuel Pérez-Sánchez, Héctor Aquiles Maldonado-Martínez, Maybelline Robles-Estrada, Enrique Bargalló-Rocha, Paula Cabrera-Galeana, Maritza Ramos-Ramírez, Yolanda Irasema Chirino, Luis Alonso Herrera, Luis Ignacio Terrazas, Cecilia Frecha, Javier Oliver, Sandra Perdomo, Felipe Vaca-Paniagua

**Affiliations:** 1 Posgrado en Ciencias Biológicas de la Universidad Nacional Autonóma de Mexico, Facultad de Estudios Superiores Iztacala, UNAM, Mexico City 54090, Mexico; mag_hector@hotmail.com; 2 Laboratorio Nacional en Salud, Diagnóstico Molecular y Efecto Ambiental en Enfermedades Crónico-Degenerativas, Facultad de Estudios Superiores Iztacala, Tlalnepantla 54090, Mexico; erarroji@gmail.com (E.R.-J.); cdiazvelasquez@aol.com (C.D.-V.); rosalia.quezadaurban@petermac.org (R.Q.-U.); fernando.vallejo.lecuon@gmail.com (F.V.-L.); literrazas@unam.mx (L.I.T.); 3 Unidad de Biomedicina, Facultad de Estudios Superiores Iztacala, UNAM, Tlalnepantla 54090, Mexico; audelacm@gmail.com (A.d.l.C.-M.); irasemachirino@gmail.com (Y.I.C.); 4 Division of Breast Cancer, Department of Medical Oncology, Mt. Sinai Hospital, University of Toronto, Toronto, ON M5G 1X5, Canada; javier.mejiagomez@mail.utoronto.ca; 5 Sir Peter MacCallum Department of Oncology, University of Melbourne, Melbourne, VIC 3000, Australia; 6 Cancer Research Division, Peter MacCallum Cancer Centre, Melbourne, VIC 3000, Australia; 7 Instituto Nacional de Cancerología, Mexico City 14080, Mexico; fany.porras@gmail.com (F.I.P.-R.); manuelps98@gmail.com (V.M.P.-S.); arzhaus@yahoo.com (H.A.M.-M.); ebargallo@yahoo.com (E.B.-R.); drapaulacabrera@gmail.com (P.C.-G.); maritzaramos1304@gmail.com (M.R.-R.); metil@hotmail.com (L.A.H.); 8 Hospital General de Pachuca SSA, Pachuca 42070, Mexico; roem1825@hotmail.com; 9 Instituto Nacional de Medicina Genómica, Mexico City 14610, Mexico; 10 Unidad de Investigación Biomédica en Cáncer, Instituto de Investigaciones Biomédicas—Instituto Nacional de Cancerología, Mexico City 14080, Mexico; 11 Unidad de Producción Celular del Hospital Regional Universitario de Málaga—IBIMA—Málaga, 29010 Málaga, Spain; frechacecilia@gmail.com; 12 Medical Oncology Service, Hospitales Universitarios Regional y Virgen de la Victoria, Institute of Biomedical Research in Malaga, CIMES, University of Málaga, 29010 Málaga, Spain; javiom@gmail.com; 13 Genomic Epidemiology Branch, International Agency for Research on Cancer (IARC/WHO), 150 Cours Albert Thomas, 69372 Lyon, France; perdomos@iarc.fr

**Keywords:** triple-negative breast cancer, tumor evolution, metastasis, lymph nodes, early divergence, targeted therapies

## Abstract

**Simple Summary:**

Triple-negative breast cancer (TNBC) is a clinically, phenotypically, and molecularly heterogeneous disease. This heterogeneity is a factor that negatively impacts therapy response. To analyze evolutionary patterns and the genomic alterations in patients with clinically aggressive disease who did not respond to treatment, we performed whole-exome sequencing in multiple longitudinal samples from diagnosis to distant metastasis. We found an extensive intrapatient and interpatient genetic heterogeneity, mutational signature composition at different stages, and, interestingly, an early lymph node metastasis formation during the evolution of aggressive TNBC. This study provides detailed insights into the genomic complexity, and the phylogenetic and evolutionary development of TNBC, as well as identifying specific mutations associated with targeted treatments in TNBC.

**Abstract:**

In triple-negative breast cancer (TNBC), only 30% of patients treated with neoadjuvant chemotherapy achieve a pathological complete response after treatment and more than 90% die due to metastasis formation. The diverse clinical responses and metastatic developments are attributed to extensive intrapatient genetic heterogeneity and tumor evolution acting on this neoplasm. In this work, we aimed to evaluate genomic alterations and tumor evolution in TNBC patients with aggressive disease. We sequenced the whole exome of 16 lesions from four patients who did not respond to therapy, and took several follow-up samples, including samples from tumors before and after treatment, as well as from the lymph nodes and skin metastases. We found substantial intrapatient genetic heterogeneity, with a variable tumor mutational composition. Early truncal events were *MCL1* amplifications. Metastatic lesions had deletions in *RB1* and *PTEN*, along with *TERT*, *AKT2*, and *CCNE1* amplifications. Mutational signatures 06 and 12 were mainly detected in skin metastases and lymph nodes. According to phylogenetic analysis, the lymph node metastases occurred at an early stage of TNBC development. Finally, each patient had three to eight candidate driving mutations for targeted treatments. This study delves into the genomic complexity and the phylogenetic and evolutionary development of aggressive TNBC, supporting early metastatic development, and identifies specific genetic alterations associated with a response to targeted therapies.

## 1. Introduction

Triple-negative breast cancer (TNBC) is defined by a lack of expression of estrogen receptors (ER), progesterone receptors (PR), and an amplification or overexpression of human epidermal growth factor receptor type 2 (HER2), and accounts for 10–15% of breast cancer cases [1,2]. TNBC is treated with chemotherapy based on anthracyclines, taxanes, and platinum salts [3]. Despite optimal management of TNBC treatment, only 30% of patients achieve a pathological complete response (pCR) with neoadjuvant chemotherapy (NAC) [3]. This differential response with NAC has been attributed, at least in part, to intratumoral heterogeneity and continuous tumor evolution, which facilitate the growth of treatment-resistant subclones and their subsequent spread through metastatic events [4,5].

Breast cancer (BC) associated mortality is a consequence of the metastatic spread of a primary tumor to different organs and accounts for 90% of all deaths from breast cancer [6]. BC has different clinical scenarios and metastatic spread patterns. Lymph nodes are the most common sites of metastases in BC, and they are associated with a bad prognosis and recurrence [7,8]. Skin metastases, on the other hand, occur in up to 20% of all cases and these are also associated with a poor prognosis [9,10]. Several studies have demonstrated that primary tumors share many mutations with their metastases, but at the same time each metastatic lesion has clear differences in the number and type of affected genes as a result of tumor evolution and selective treatment pressures [5,11,12,13].

Usually, primary tumors and metastases are the most available samples [5,11,12,14]. However, studying evolution in TNBC has been challenging due to the difficulty of obtaining samples at all stages of the disease. In this context, we performed whole-exome sequencing (WES) on 16 samples from four patients with clinically aggressive disease who did not respond to several lines of treatment in order to analyze the evolutionary patterns of the cancerous cells, and to describe their genomic allelic composition and mutational patterns. Our analysis included a set of follow-up samples, such as tumor samples (before and after treatment), as well as samples from lymph nodes, skin metastases, and normal tissue. Our data show an extensive intra- and interpatient genetic heterogeneity. Additionally, these data indicate an early divergence to lymph nodes during TNBC carcinogenesis. Finally, we identified potential candidate genes that could be suitable for targeting with therapeutic precision agents which may contribute to preventing treatment failure in the future.

## 2. Materials and Methods

### 2.1. Sample Information

A total of 97 patients were diagnosed with TNBC and treated at the National Cancer Institute (INCAN) of Mexico between April 2007 and April 2010. TNBCs were diagnosed by immunohistochemistry (IHC) based on the criteria of less than one percent expression of the staining score for the ER, PR, and HER2 receptors. We selected cases with clinically aggressive disease, defined as the absence of pCR to NAC, a lack of response to any treatment, death within three years of diagnosis, and with ductal carcinoma (Table S1). After DNA quality control, only 16 samples from four patients were amplifiable and suitable for WES analysis, including primary treatment-naïve tumors, treated tumors, lymph nodes, and skin metastases, all of which were paired with their respective normal samples. For this study, the protocol was approved by the Research and Ethics Committees (protocol #016/013/IBI CEI/1021/16) and conducted by the Declaration of Helsinki.

### 2.2. Sample Preparation and DNA Extraction

Samples were obtained from formalin-fixed paraffin embedded (FFPE) tissues and extracted from three 50 μm slices corresponding to about 50–200 mg of tissue. Samples were dewaxed with xylol and ethanol baths and digested overnight with proteinase K. Crosslinking formation by formaldehyde was reversed through a 90 °C incubation for 1 h. DNA extraction was done with the DNeasy Blood & Tissue Kit (Qiagen, Hilden, Germany), following the manufacturer’s instructions. DNA concentration was quantified by Qubit (Invitrogen, Carlsbad, USA). DNA integrity and purity were verified by agarose gel electrophoresis and spectrophotometry, respectively, to evaluate DNA quality.

### 2.3. Library Preparation

The library preparation was done on the Illumina Nextera Rapid Capture Exome Kit v.1.2 (Illumina, San Diego, CA, USA), which covers 37 Mb of protein-coding bases, following the manufacturer’s instructions. Pair-end sequencing was performed on an Illumina HiSeq 2500 for 2 × 150 cycles, at *the Red de Apoyo a la Investigación* of the National Institute of Nutrition Salvador Zubirán, Mexico City, Mexico.

### 2.4. SNV e Indels Identification

Sequencing reads were aligned to the human genome reference hg19 with BWA-MEM [15]. GATK tools were used to sort reads, mark duplicates, and for base recalibration [16]. Single nucleotide variations (SNV) were called with Mutect2 [17] and annotated with ANNOVAR [18]. Variants were filtered as follows: (i) variants with an allelic frequency of less than 0.001 in 1000 genomes, ExAC and ESP6500 databases, were included; (ii) common SNP were excluded, unless they were reported as pathogenic in ClinVar; (iii) variants with at least two reads in both DNA chains in the tumor samples were kept; and (iv) variants with an allelic fraction of ≥0.05 were also kept. We used ExAC to exclude non-pathogenic natural variation, considering that this repository includes the sequencing data of 5789 exomes of Latin and admixed Americans.

Driver genes were identified using two well-curated gene lists: Intogen v.2019 (https://www.intogen.org/) and the list from Yates [5]. Then, driver mutations were defined as pathogenic if they were previously described in ClinVar as pathogenic, if at least two out of three algorithms—SIFT, PolyPhen2 or MutationTaster—predicted them as deleterious, and if they were known hotspot mutations in COSMIC [19] and cBioportal [20] (Table S2). All driver variants were manually curated by inspection of the BAM files using the IGV software [21].

### 2.5. Tumor Mutational Burden Analysis

Tumor mutational burden (TMB) was defined as the number of somatic, coding, and indel mutations, and base substitutions per megabase of the examined genome. To calculate the TMB per megabase, the total number of counted mutations was divided by the size of the coding region of the targeted territory. An estimated exome size of 37 Mb was considered for the analyses.

### 2.6. CNV Identification

Copy number variation (CNV) was identified with CNVkit [22], using a panel of normal samples with default parameters. We defined the driver genes according to the Intogen and Yates lists previously described. The found variants were filtered by directionality, considering the biology of the affected gene as follows: (i) CNV with CN = 2 were excluded; (ii) oncogenes with copy number (CN) < 2 were excluded and CN > 2 were kept; (iii) tumor suppressor genes with CN > 2 were excluded and CN < 2 were kept.

### 2.7. Mutational Signature Analysis

Mutation patterns were evaluated in the context of 96 trinucleotides using the R package deconstrictSigs [23]. To evaluate the distribution of the samples with mutational signatures, an unsupervised hierarchical clustering analysis was performed by calculating Euclidean distances.

### 2.8. Pathway Enrichment Analysis

Pathway and network enrichment analysis was defined with the David Functional Annotation Tool 6.8 [24], using driver genes in the KEGG pathway database. Uninformative pathways were eliminated, such as general cancer and non-cancer diseases.

### 2.9. Global Actionable Alterations

Treatments approved by the FDA for actionable genes in all types of cancer were selected using the OncoKB database [25].

### 2.10. Evolutionary Tree Construction

Phylogenetic trees were constructed with SNV, indels, and CNV, using maximum parsimony principles [26]. Variants were coded with 0 and 1 to indicate the absence and presence of mutations, respectively, and processed in mesquite (http://www.mesquiteproject.org/) to generate nexus files. The phylogenetic trees were analyzed in PAUP (http://paup.phylosolutions.com/) using a heuristic method and rooted with normal samples. The reliability of trees was evaluated with the Bootstrap Method [27]. The trees generated were saved in Newick format and visualized with the FigTree software (http://tree.bio.ed.ac.uk/software/figtree/).

## 3. Results

### 3.1. Patients with TNBC Exhibit a Variety of Clinical Scenarios

**Figure 1:**
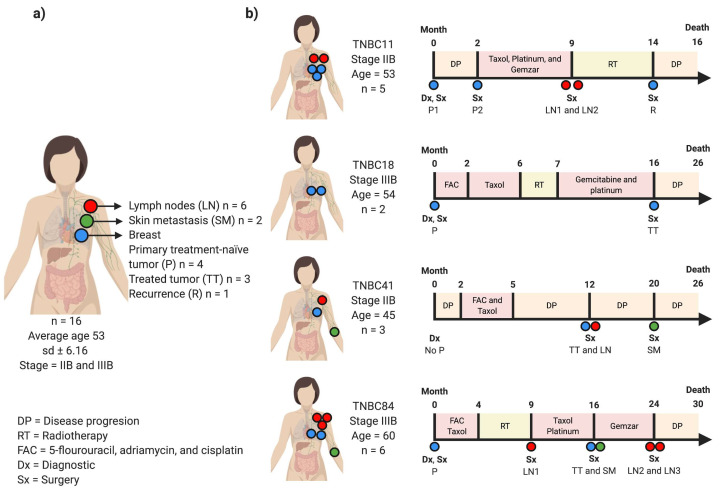
Clinical history of TNBC patients. (**a**) The cohort of TNBC with 16 samples from four patients includes tumors obtained before and after treatments, lymph node- and skin metastasis. (**b**) The timeline depicts the clinical characteristics of all patients analyzed and samples taken during the progression of the disease. Colored boxes show the different treatments received by patients (blue: breast; red: lymph node metastases; green: skin metastases).

For this study, we reviewed the database of patients diagnosed with TNBC and treated at the INCAN during April 2007 and April 2010. We chose 16 FFPE samples from four patients with aggressive disease and high-quality DNA to perform WES. Four clinical settings were represented by the samples: (i) primary treatment-naïve tumor, (ii) treated tumor, (iii) lymph nodes, and (iv) skin metastasis. All patients were diagnosed at advanced stage and received NAC treatment. None of them achieved a pCR and all died within three years of their diagnosis (Figure 1a,b; Table S1).

**Figure 2:**
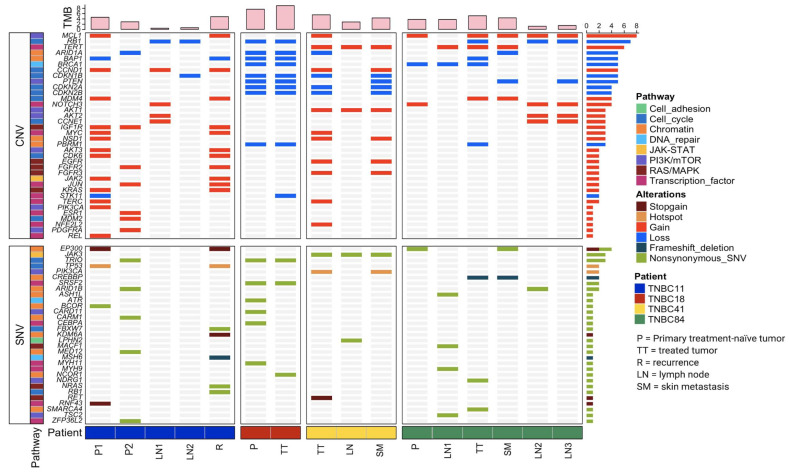
Allelic composition and CNV alterations in driver genes. The samples were segmented and color-coded by patient and sorted chronologically. TMB is indicated on the top panel with pink bars. Driver genes are classified into two categories: SNV (lower panel) and CNV (upper panel). Mutation signaling pathways in which driver genes are involved, as well as mutation types, are denoted by distinct colors.

Patient TNBC11 was a woman diagnosed at age 53 with a locally advanced TNBC. A first biopsy of the primary tumor was obtained and had pathogenic variants in *TP53* and *EP300*, as well as amplifications in *MCL1* and *IGF1R* (Figure 1 and Figure 2). Then, a second biopsy was taken from the same primary tumor at month two, and the patient started chemotherapy treatment. This second sample had different driving alterations and only shared the *IGF1R* amplification with the previous sample. At the ninth month, a mastectomy was performed, and two lymph node biopsies were obtained. Both lymph nodes showed *RB1* deletions and low TMB. Lymph node 1 showed *NOTCH3*, *AKT2*, and *CCNE1* amplifications, and lymph node 2 had a CNV loss in *CDKN1B*. At month 14, after five months of radiotherapy, a last mastectomy was performed and a recurrence sample was obtained, which showed a composition of the altered driver genes very similar to those detected in the primary tumour 1, including the *EP300* and *TP53* mutations, and amplifications in *MCL1*, *CCND1*, *MDM4*, *IGF1R*, *MYC*, *AKT3*, *CDK6*, *JAK2*, *KRAS*. The recurrence also shared a *BAP1* deletion with the primary tumor 1. Globally, all the neoplastic lesions had 57 driver alterations. Patient TNBC11 died after the 16th month of diagnosis.

Patient TNBC18 was a woman diagnosed at age 54 with two available samples, a primary treatment-naïve tumor and a treated tumor. The primary treatment-naïve tumor was obtained in the first month of diagnosis. After 16 months of treatment with chemo- and radiotherapy, surgery was performed to obtain the treated tumor. Both samples showed a very similar pattern of alterations and a high TMB. Nine deletions were shared, including *RB1*, *ARID1A*, *BAP1*, *BRCA1*, *CDKN1B*, *PTEN*, *CDKN2A*, *CDKN2B*, and *PBRM1*. Patient TNBC18 had a total load of 28 driver alterations and died in the 26th month of diagnosis.

Patient TNBC41 was diagnosed at age 45 and had three samples taken from a treated tumor, a lymph node and a skin metastasis. The primary treatment-naïve tumor was not available. After 12 months of treatment with chemotherapy, a mastectomy was performed, and two samples were obtained: a treated tumor and a lymph node metastasis. The treated tumor showed several CNVs in driver genes, including amplifications in *MCL1*, *TERT*, *CCND1*, *AKT1*, *MYC*, *EGFR*, and *FGFR3*; deletions in *CDK1B*, *CDKN2A* and *CDKN2B*; a *PIK3CA* hotspot mutation; and a high TMB. In contrast, the lymph node metastasis had only four pathogenic alterations and a lower TMB. In the 20th month, further surgery was carried out, and a skin metastasis biopsy was obtained. The skin metastasis sample showed a very similar pattern of alterations in driver genes as compared with the treated tumor, including the *PIK3CA* hotspot mutation, the amplifications in *CCND1*, *EGFR*, and *FGFR3*, and the deletions in *CDK1B*, *CDKN2A,* and *CDKN2B*. This skin metastasis also had a private *PTEN* deletion. The three samples shared SNV in *JAK3* as well as *TERT*, and *AKT1* amplifications. Patient TNBC41 had a total number of 33 driver genes affected and died in the 26th month after diagnosis.

Patient TNBC84 was diagnosed at age 60 and had six samples taken. The primary treatment-naïve tumor was extracted in the diagnosis and had *MCL1* and *NOTCH3* amplifications, and a *BRCA1* deletion. After nine months of chemo- and radiotherapy, surgery was performed, and a lymph node biopsy was obtained. This had a *TERT* amplification, a *BRCA1* CNV loss, and a *TSC2* pathogenic mutation. A treated tumor and a skin metastasis sample were obtained in the 16th month, and both showed *MCL1*, *TERT,* and *MDM4* amplifications. Finally, additional surgery was performed in the 24th month, and samples were obtained from two lymph nodes: lymph nodes 2 and 3. Both lymph nodes showed *RB1* deletions, as well as *MCL1*, *NOTCH3*, *AKT2,* and *CCNE1* amplifications. Interestingly, all the lesions excluding lymph node 1 had *MCL1* amplifications. Patient TNBC84 harbored 39 driving alterations and died in the 30th month after diagnosis.

### 3.2. Patients with TNBC Display an Extensive Genetic Heterogeneity

Our sequencing analysis identified an extensive genetic interpatient heterogeneity. We found a total of 2264 SNVs in the 16 samples, including 611 synonymous, 1539 non-synonymous mutations, and 114 indels. TMB was broad, ranging from 0.4–8.9 mutations per megabase, with an overall mean of 3.8 (Figure 2). Each patient’s sample showed a unique TMB. For instance, for patient TNBC11 the recurrence sample had the highest TMB, while for patients TNBC18, TNBC41, and TNBC84 the highest TMB was found in samples coming from a treated tumor. On the other hand, lymph nodes showed the lowest TMB in three out of four patients analyzed (TNBC11, TNBC41, and TNBC84).

We identified 157 mutations in 67 driver genes, from which 114 were CNV (median 6.5, range 2–15 for each sample) and 43 were SNV (median 2.5, range 0–7 for each sample). The full list of driving mutations is presented in Tables S2 and S3. The most prevalent alterations in our cohort were CNVs, *MCL1* (50%), *RB1* (43.75 %), *TERT* (37.5%), *ARID1A*, *BAP1*, *BRCA1*, *CCND1*, *CDKN1B*, and *PTEN* (31.25%) being the most frequently affected genes.

The gene ontology analysis revealed enrichment in eight important signaling pathways: transcription factors (22.4%), chromatin (20.9%), cell cycle (19.4%), PIK3/mTOR (16.4%), RAS/MAPK (11.9%), JAK-STAT (2.9%), and cell adhesion (1.5%). Most of the driver genes and signaling pathways were shared by patients TNBC18 (primary treatment-naïve tumor and treated tumor) and TNBC41 (treated tumor and skin metastases). In contrast, the most heterogenous samples were TNBC11 and TNBC84, which shared only a few signaling pathways (Figure 2).

### 3.3. Mutational Processes Act on Different Stages of TNBC

**Figure 3:**
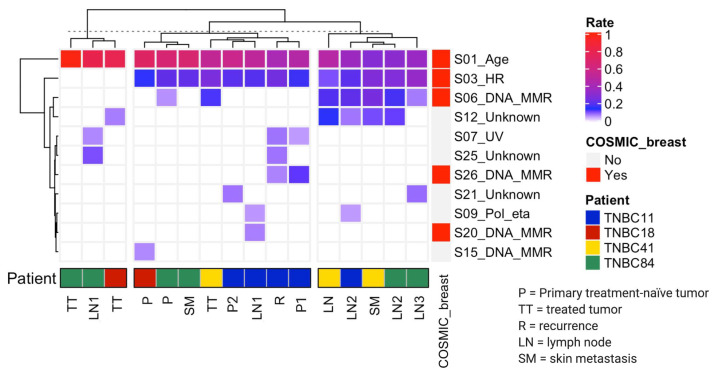
Mutational signature profile in TNBC patients. The samples are ordered according to the hierarchical grouping by mutational signature composition described above. The band of COSMIC breast describes the signatures found in the database of COSMIC Sanger, and the color palette shows the frequency of signatures in each sample. Rate is expressed as the relative frequency of the mutations associated with the corresponding signature. Mutational signatures previously identified in breast cancer samples in the COSMIC database are shown in red boxes. Patients are color-coded as in Figure 2. The tissue origin of the sample is indicated.

To identify the mutational processes acting on TNBC, a mutational signature analysis using deconstrictSigs in the R language was performed. Eleven of the 30 mutational signatures previously described by Alexandrov [28] were detected with a median of 3.5 per sample (range 1–5). Five of these signatures have been previously identified in BC, while the remaining six are identified for the first time in BC patients (Figure 3). Signature S01 (age-related) was prevalent in all samples and contributed the highest mutational rate. Signature S03 (homologous recombination deficiency) was detected in 81% of the samples, S06 (DNA mismatch repair deficiency) was detected in 44%, and S12 (unknown etiology) in 31%. Other signatures were found in fewer than 20% of the samples.

To identify potential common mutational processes active between all samples, we performed an unsupervised hierarchical clustering analysis, calculating Euclidean distances to determine the sample distribution of mutational signatures. This analysis classified the samples into three groups: (i) samples sharing a single mutational signature (3/16), (ii) those sharing two (8/16), and (iii) those sharing three or more (5/16) mutational signatures. Each group had unique characteristics. For instance, group two shared two mutational signatures (S01 and S03), albeit in different types of samples for each patient. On the other hand, group three includes signatures S01 and S03, but also acquired two additional signatures, S06 and S12. Interestingly, this group included lymph node and skin metastasis samples from three patients (TNBC11, TNBC41, TNBC84).

### 3.4. Early Divergence in TNBC

**Figure 4:**
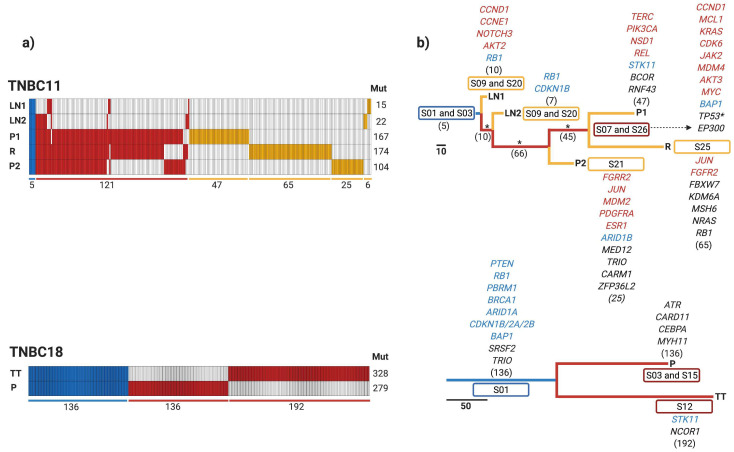

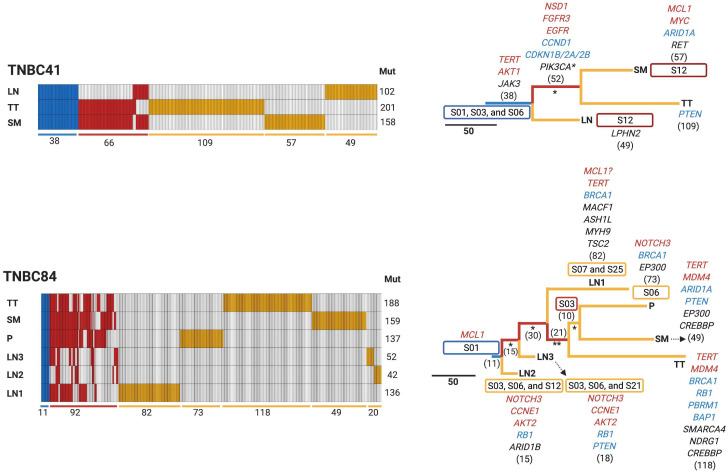
Phylogenetic trees of TNBC patients. (**a**) Heatmap depicts truncal (blue), shared (red), and private (yellow) mutations found in each sample. The number of mutations is shown at the bottom, and the total mutations for each sample are placed on the right. (**b**) Phylogenetic trees depict the relationship of samples from each patient. The length of the branches is proportional to the number of detected mutations. Driver genes such as SNV (black), amplifications (red), and deletions (blue), as well as mutational signatures found, are shown for each sample in this cohort. P: primary treatment-naïve tumor; TT: treated tumor; R: recurrence; LN: lymph node; and SM: skin metastasis. * = bootstrap value >95%, and ** = bootstrap value >65% and <95%.

To determine the evolutionary history of the TNBC tumors, a phylogenetic analysis was performed. We classified SNV, indels, and CNV into three groups: (i) truncal (mutations found in all samples coming from the same patient), (ii) shared (mutations discovered in more than one sample of the same patients but not in all of them), and (iii) private (mutations found only in one sample of the same patient) (Figure 4a). A maximum parsimony algorithm [26] and the nonparametric bootstrapping methods [27] were used to estimate tumor phylogenies.

Each patient had a unique combination of truncal, shared, and private mutations, but we could observe that recurrence and treated tumors exhibited the highest number of private mutations, and lymph nodes had the fewest private mutations. These findings show a large genetic intrapatient heterogeneity distributed in all the neoplastic lesions. For example, TNBC11 had five truncal mutations, 121 shared, and each sample had a different number of private mutations, with the recurrence sample containing the most private mutations. Patient TNBC18, on the other hand, had 136 truncal mutations, with 136 and 192 private mutations in the primary treatment-naïve tumor and treated tumor, respectively. TNBC41 had 38 truncal mutations, 66 shared, and the treated tumor was the sample that had the most private mutations (109). TNBC84 contained 11 truncal mutations, 92 shared, and the treated tumor was the most mutated, showing 118 mutations.

Based on the phylogenetic tree, three patients (TNBC11, TNBC41, and TNBC84) had the same evolutionary patterns (Figure 4b). Primary treatment-naïve tumor, treated tumor, recurrence, and skin metastasis samples were all identified in the same clade and shared the most recent common ancestor (MRCA). Skin metastasis and primary treatment-naïve tumor from TNBC84 were clustered in the same clade since these two samples shared numerous mutations. Interestingly, lymph nodes were clustered together suggesting an early divergence event in TNBC patients. On the other hand, lymph nodes had fewer driver genes, possibly indicating an evolutionary bottleneck. Notably, we could identify 136 truncal mutations in the two samples obtained from TNBC18. Unfortunately, we could not evaluate the evolutionary patterns of this patient due to the low number of samples available.

### 3.5. Potential Clinical Implications of Mutations Detected in TNBC

**Figure 5:**
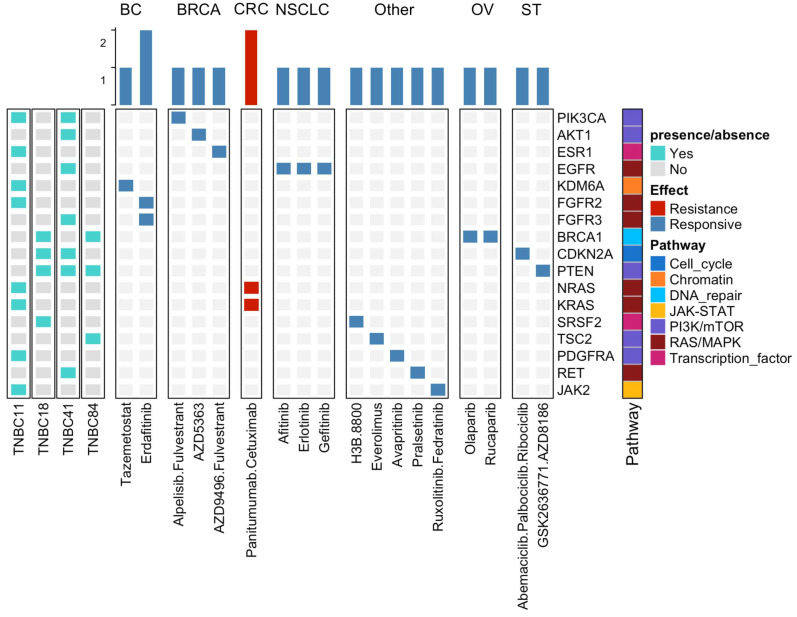
Actionable alterations in TNBC according to the oncoKB database. The figure depicts the actionable alterations found in our study and the corresponding FDA-approved drug. The drugs were segmented according to tumor type. Signaling pathways are indicated on the right and the patient identification on the left. BC: Bladder Cancer; BRCA: Breast Cancer; CRC: Colorectal Cancer; NSCLC: Non-Small Cell Lung Cancer; OV: Ovarian Cancer; and ST: Solid Tumors.

A specific search in the precision oncology knowledge base OncoKB [25] was performed to identify specific mutations associated with treatment response. Of note, TNBC is almost exclusively treated with chemotherapy [3]. Interestingly, we could identify 17 targets (3–8 targets per patient), distributed in seven signaling pathways. RAS/MAPK and PIK3/mTOR were the two signaling pathways with the highest number of actionable targets (Figure 5).

When we clustered actionable alterations based on detected mutations by tumor type, we observed seven distinct groups of tumors and their associated treatments. For instance, TNBC patients bearing mutations in *PIK3CA*, *AKT1,* and *ESR1*, as well as *EGFR* amplifications, may be treated with different drugs to those that are used to treat other subtypes of breast cancer (BRCA) and Non-Small Cell Lung Cancer (NSCLS), respectively. This helps to open new therapeutic windows in TNBC based on the potential candidate genes suitable for therapeutic precision agents.

## 4. Discussion

The purpose of this study was to characterize the molecular features and evolution of tumors in patients with clinically aggressive TNBC. TNBC shows the worst prognosis of all breast cancers, with still unrevealed molecular aspects. We chose four patients from whom several follow-up samples were taken. We used a WES approach to characterize tumor heterogeneity, mutational signature profiles, driver genes, and tumor evolution in a total of 16 samples. Each patient was diagnosed at an advanced stage and presented a unique clinical scenario. These scenarios are in line with the complexity of this neoplasm and the difficulty for the development of specific treatments, given the wide genetic heterogeneity observed among patients [3]. In our cohort none of the patients achieved a complete response with NAC and all of them died within three years. Our findings are consistent with and similar to previously reported mortality rates in Latin America and worldwide [29,30,31].

In addition, we discovered an extensive inter- and intrapatient genetic heterogeneity in terms of CNV and SNV, in line with previous studies [5,13,32,33,34]. SNV in *TP53*, found in the primary treatment-naïve tumor in TNBC11, and *MCL1* amplifications in the primary treatment-naïve tumors in TNBC11 and TNBC84, suggest that these alterations are early events in TNBC carcinogenesis. *TP53* is the most frequently encountered SNV in TNBC and is considered to occur early in TNBC carcinogenesis [5,32]. Conversely, *MCL1* amplifications have been associated with resistance to neoadjuvant chemotherapy and are frequently enriched in late-stage patients [35], which may explain why TNBC11, TNBC41, and TNBC84 patients did not achieve a PCR, although other alterations not detected in this study cannot be ruled out.

**Figure 6:**
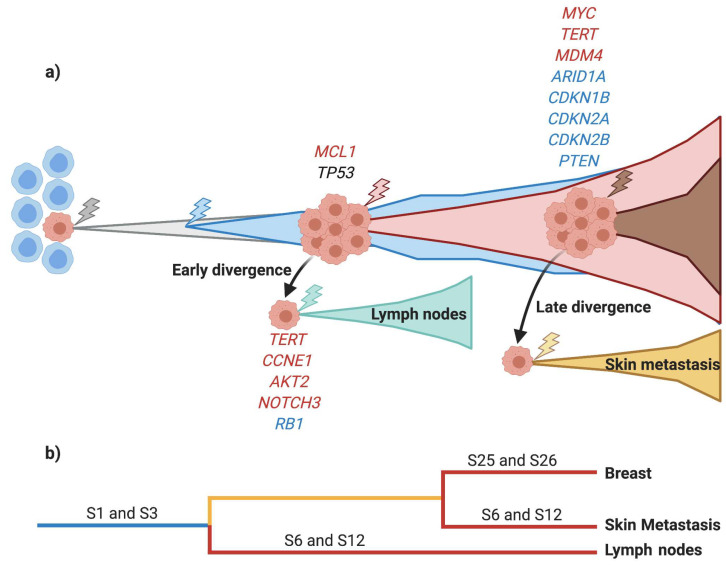
Summary of the key conclusions from our study. (**a**) Tumor evolution of TNBC seen in our cohort. We identified an early and late divergence to lymph nodes and skin metastasis, respectively. Driver genes with SNV (black), amplifications (red), and deletions (blue) are shown at each stage of TNBC carcinogenesis. (**b**) Phylogenetic tree of mutational signatures shows the main signatures acquired in each sample. Truncal mutational signatures are indicated in blue, shared signatures in yellow, and private signatures in red.

Deletions in *RB1* were predominantly found in the lymph nodes of two patients (TNBC11 and TNBC84) and deletions in *PTEN* were found in three (TNBC18, TNBC41 and TNBC84) of four patients, primarily in skin metastasis. These findings are similar to those of Bertucci et al., who identified five genes, including *RB1* and *PTEN*, enriched in metastatic TNBC [36], in addition to *ARID1A* alterations that were found to be enriched in metastatic TNBC lesions [14]. *TERT* amplifications were detected in treated tumors, lymph nodes, and skin metastasis of two patients (TNBC41 and TNBC84), as well as *AKT2* and *CCNE1* amplifications in lymph nodes of two patients (TNBC11 and TNBC84). These CNVs have been reported in other studies of advanced and metastatic breast cancer. For example, Watkins et al. found that *TERT* and *CCNE1* were subclonal and appeared at intermediate and late stages during the progression of 22 tumors, including breast cancers [37]. Overall, TNBC exhibited an extensive genetic heterogeneity, with numerous genes involved in various stages of TNBC carcinogenesis (Figure 6a).

TNBC patients showed a heterogenous TMB, with an average of 3.8 mutations (range 0.4–8.9). These results are comparable to other studies [31,38]. The treated tumors and recurrences in our cohort had the highest TMB in comparison to primary treatment-naïve tumor, lymph node, and skin metastasis samples. These TMB differences among tumor sites may be a result of tumor development per se, or the effect of neoadjuvant chemotherapy. In fact, it has been demonstrated that neoadjuvant chemotherapy alters the genetic landscape of tumors and promotes disease progression by increasing TMB in non-responder patients [39,40]. By contrast, lymph nodes show the lowest TMB, which may be an effect of an evolutionary bottleneck during cancer cell spread due to the tissue specific environmental constrains, a reduced number of invading cells (low genetic diversity), or reduced cellularity in these samples.

The analysis of mutational signatures revealed the presence of signature S01 in 100% of samples, S03 in 83%, S06 and S12 in less than 50%, and other signatures in less than 20% of samples. Unsupervised hierarchical clustering analysis based on Euclidean distances identified three groups of samples according to their common mutational signatures. Group two shared two signatures, S01 and S03, each of them detected in various tumor samples. Conversely, group three was the one showing more shared signatures, namely, S01, S03, S06, and S12. Metastatic lesions from lymph nodes and skin were the hallmarks in group three. In general, the mutational signature analysis revealed that advanced lesions are influenced by a more complex mutational environment. Five out of the 11 mutational signatures identified in our cohort had been associated with breast cancer, including S01, S03, S06, S20, and S26 [41], while the remaining six had not been detected in breast cancer until now. We discovered that certain signatures are active at various stages of the disease. Indeed, S01 and S03 were found in almost all samples, whereas S06 and S12 were found in lymph node and skin metastasis samples (Figure 6b). Mutational signature S01 is frequent in all type of tumors due to its association with spontaneous 5-methyl cytosine deamination accumulated with age. S03 has been found in at least 20% of all molecular types of breast cancer; however, in TNBC this signature constitutes more than 70% of the cases [41,42]. The mutational signatures S06, S20, and S26, associated with mismatch repair deficiency, were detected in our study, and are also prevalent in breast cancer [41,42]. Other studies, like the one from Gerstung et al., also reported that signatures associated with DNA mismatch repair deficiency were developed in the late stages of 38 different types of cancer, including breast cancer [34], and were associated with high-grade breast tumors [43]. Although there is no for evidence for the S12 signature in breast cancer, this signature has been detected in liver cancer, accounting for a small proportion of the total number of mutations (approximately 20%) found in this neoplasm. On the other hand, patients not achieving pCR after platinum-based NAC have been reported to exhibit some signatures associated with platinum exposure [39], including S04, S24, and S29, but these signatures were not observed in our cohort. Nevertheless, we do not rule out the prevalence of signatures associated with resistance to platinum-based NAC, which could be evidenced by whole genome sequencing.

Breast cancer cells spread to other tissues through hematogenous and lymphatic routes [44]. Understanding evolutionary patterns is fundamental to improving the comprehension of the progression of tumors and metastatic invasion to other tissues. In this study, we observed a defined evolutionary pattern in three out of four patients analyzed. The primary treatment-naïve tumor, treated tumors, recurrences, and skin metastases formed clusters in the same clade, indicating that they shared an MRCA. It has been hypothesized for many years that axillary lymph nodes constitute a reservoir for further dissemination to distant organs [45,46]. However, our study showed that skin metastasis was seeded directly from the primary treatment-naïve tumor, regardless of lymph node stage. In addition, lymph nodes formed an independent clade with a few mutations shared between the primary treatment-naïve tumor and other lesions, suggesting that spreading may occur early in these tissues. This same pattern has been reported in other molecular studies [13,32,47]. Clinical and molecular analyses in TNBC and breast cancer have also shown dissemination to lymph nodes from small T1 tumors (<2 cm), and the occurrence of metastatic spread in the early stages of tumor growth [48,49,50]. On the other hand, lymph nodes had fewer altered driver genes, indicating an evolutionary bottleneck. This bottleneck may be caused by the seeding of a small number of invasive cells into lymph nodes, which may result in a loss of genetic diversity. Reduced clonal heterogeneity in metastatic lymph nodes after NAC has been reported in breast, colorectal, and lung cancer patients [49]. Taken together, these findings support the early metastasis model and imply a rapid metastatic progression from early lesions in those patients with aggressive disease [51].

The only current systemic treatment approved for TNBC patients is chemotherapy. Given the high degree of genetic heterogeneity in TNBC, it is possible to use this molecular trait to identify candidates for targeted therapy. We detected 17 potential actionable alterations clustered in seven altered signaling pathways, which are consistent with previous findings [5,31,52]. We identified seven groups of tumors and their associated treatments based on the type of mutations detected; these treatments are also used in other subtypes of breast cancer and other tumor types. Indeed, our analysis identified potential valuable genetic alterations that could be used in the treatment of TNBC.

Our study provides a detailed analysis of four patients with TNBC. The cancers were characterized molecularly and the wide genetic heterogeneity they showed may be impacting on the clinical treatment and the evolution of this disease. However, the limitations of this study include: the impossibility to correlate clinical and molecular features due to sample size limitations; and the fact that the cases were not homogenous in terms of time of progression and treatment, as each patient exhibited unique clinical characteristics and different stages of the disease, and so the treatments were heterogeneous. Another limitation of this study is that its design does not allow an evaluation of the role of ethnicity in the evolution of the disease. In addition, the use of FFPE samples and a potential low tumor cellularity in lymph node samples may have hampered the identification of the totality of mutations in these tissues. The clinical relevance of using WES in non-pCR patients longitudinally may allow an evaluation of the prevalence and composition of genetic driver alterations that can be used as potential actionable targets, and which evolve as resistance mechanisms. However, the lack of a pCR group limited the comparison of the evolution of tumors with better prognoses.

Despite these limitations, our data further supports previous findings from other studies and evidences the notorious intrapatient genetic heterogeneity associated with all tumor lesions, as well as elucidating the molecular architecture and the genomic evolution of these aggressive neoplasms. The performed longitudinal analysis enabled the establishment of phylogenetic relationships between patients’ neoplasms and the identification of an early divergency in lymph nodes. The mutational signatures S06 and S12 were found to be exclusive to lymph nodes and skin metastasis, and, finally, actionable alterations were identified for TNBC treatment. To our very best knowledge, this is the first study that integrates genomic alterations with evolutionary processes in a TNBC cohort from Latin America, which is, in turn, an underrepresented ethnicity in most genomic studies. Taken together, these findings suggest that the extensive intrapatient genetic heterogeneity found in all malignant lesions may play a determinant role in the therapeutic failure, evolutionary adaptability, and aggressive clinical behavior of TNBC.

## 5. Conclusions

This study provided detailed insights into the genomic complexity and intrapatient heterogeneity, as well as the phylogenetic and evolutionary development of TNBC. It also identified genomic features that have been associated with beneficial targeted treatments. We integrated the comprehensive genomic alterations with evolutionary techniques to understand the evolution and the genetic variability, as well as the description of each stage of the disease progression in the TNBC patients longitudinally. These findings contribute to the elucidation of the global phenomenon of tumor evolution in TNBC and provide genomic data that may aid future studies in defining actionable alterations for these patients, thereby enabling a more personalized therapy.

## Data Availability

The data presented in this study are available in the article and Supplementary Material. Raw data is available upon request.

## Supplementary Materials

The following are available online at https://www.mdpi.com/article/10.3390/cancers13205091/s1, Table S1: Clinical characteristics; Table S2: Driver SNV; Table S3: Driver CNV.
